# Versatile and Resistant Electroless Pore-Plated Pd-Membranes for H_2_-Separation: Morphology and Performance of Internal Layers in PSS Tubes

**DOI:** 10.3390/membranes12050530

**Published:** 2022-05-18

**Authors:** David Martinez-Diaz, Valeria Michienzi, José Antonio Calles, Raúl Sanz, Alessio Caravella, David Alique

**Affiliations:** 1 Department of Applied Mathematics, Science and Engineering of Materials and Electronic Technology, Rey Juan Carlos University, 28933 Móstoles, Spain; david.martinez.diaz@urjc.es; 2 Department of Computer Engineering, Modelling, Electronics, and Systems Engineering (DIMES), University of Calabria, 87036 Rende, Italy; valeriamichienzi@gmail.com; 3 Department of Chemical, Energy and Mechanical Technology, Rey Juan Carlos University, 28933 Móstoles, Spain; joseantonio.calles@urjc.es; 4 Department of Chemical and Environmental Technology, Rey Juan Carlos University, 28933 Móstoles, Spain; raul.sanz@urjc.es

**Keywords:** palladium, electroless plating, composite membrane, intermediate layer, PSS, internal surface, hydrogen, concentration-polarization, CO inhibition

## Abstract

Pd-membranes are interesting in multiple ultra-pure hydrogen production processes, although they can suffer inhibition by certain species or abrasion under fluidization conditions in membrane reactors, thus requiring additional protective layers to ensure long and stable operation. The ability to incorporate intermediate and palladium films with enough adherence on both external and internal surfaces of tubular porous supports becomes crucial to minimize their complexity and cost. This study addresses the incorporation of CeO_2_ and Pd films onto the internal side of PSS tubes for applications in which further protection could be required. The membranes so prepared, with a Pd-thickness around 12–15 μm, show an excellent mechanical resistance and similar performance to those prepared on the external surface. A good fit to Sieverts’ law with an H_2_-permeance of 4.571 × 10^−3^ mol m^−2^ s^−1^ Pa^−0.5^ at 400 °C, activation energy around 15.031 kJ mol^−1^, and complete ideal perm-selectivity was observed. The permeate fluxes reached in H_2_ mixtures with N_2_, He, or CO_2_ decreased with dilution and temperature due to the inherent concentration-polarization. The presence of CO in mixtures provoked a higher decrease because of a further inhibition effect. However, the original flux was completely recovered after feeding again with pure hydrogen, maintaining stable operation for at least 1000 h.

## 1. Introduction

Hydrogen is receiving great attention as a suitable and clean energy vector to facilitate the energy system transition towards a zero-carbon energy future thanks to its efficient conversion into energy for multiple applications, covering the necessities of transport, industrial, and residential demands with limited CO_2_ emissions depending on the particular production strategy [1]. In this context, it is important to note that hydrogen can be generated from a wide variety of sources, including both renewable and non-renewable feedstock, thus varying the associated CO_2_ emissions and final production costs. Currently, most of the hydrogen demand is covered by production from fossil fuels, particularly via natural gas steam reforming, resulting in a considerable amount of carbon dioxide emissions and being denoted as grey hydrogen [2]. These CO_2_ emissions can be captured for storage or utilization by any in situ or, most frequently, downstream process, thus identifying it as blue hydrogen [3].

However, the most interesting routes require the promotion of carbon-free technologies to move towards green hydrogen separated from any CO_2_ emission, here including water electrolysis with electricity coming from wind or solar power, photocatalysis, and biomass gasification, among others [4]. High-purity hydrogen cannot be directly produced through thermochemical routes, which represents one of the most interesting options to reduce the carbon footprint and take advantage of mature and well-known technologies while reaching further developments and cost reduction with other greener alternatives. In these cases, the generated hydrogen is typically accompanied by nitrogen, carbon monoxide, or carbon dioxide, among others, thus requiring further purification steps [5]. This hydrogen purification can be mainly carried out by using absorption amine-based methods, pressure swing adsorption (widely known as PSA units), or membrane-based technologies [6]. During the last few decades, the use of membrane-based technologies has received growing interest due to their lower energy requirements, similar performance in both centralized and small decentralized hydrogen production centers, and the possibility of being integrated with chemical reactions in membrane reactors. The last strategy typically allows an increase in the reactant conversion, uses milder operating conditions, and intensifies the overall process. Particularly, metallic membranes based on palladium or palladium-based alloys have demonstrated a good performance for these applications, reaching an ultra-high purity of hydrogen [7,8,9].

Hydrogen permeation through these Pd-based membranes is typically described through Sieverts’ law (Equation (1)), in which the permeate flux ($J_{H_{2}}$) is directly proportional to the membrane permeability (*π’*) and the square root of the hydrogen partial pressure between retentate ($P_{H_{2},\;ret}^{0.5}$) and permeate ($P_{H_{2},\;perm}^{0.5}$) sides, but inversely proportional to the membrane thickness (*t*):

(1) $$J_{H_{2}}=\frac{\pi^{\prime }}{t}\left(P_{H_{2},\;ret}^{0.5}-P_{H_{2},\;perm}^{0.5}\right)=\pi \left(P_{H_{2},\;ret}^{0.5}-P_{H_{2},\;perm}^{0.5}\right)$$

The reduction in the metal thickness as much as possible is one of the most interesting strategies to maximize the membrane performance in terms of permeate flux, increasing its permeance value (π). However, it could compromise its mechanical resistance against moderate pressure driving forces. Moreover, taking into account the scarcity of this precious metal, the strategy also aims to reduce the overall cost of the membrane [10]. In this context, most researchers propose the preparation of composite membranes, in which a thin Pd film of only a few micrometers, which ensures a relatively low cost and high permeation capacity, is placed onto the surface of a porous substrate that provides the required mechanical resistance to the membrane [11]. These supports are usually made of alumina or porous stainless steel, providing different geometries, porosities, and surface properties that could affect the adherence and required final thickness of the palladium film to ensure a high membrane selectivity [12]. Among them, metal supports are preferred by the industry due to their facility to be handled and better fit in most current devices, typically made with carbon or stainless steel [13]. However, independently of the commercial quality, their original surface is rougher and presents larger pore mouths in comparison with ceramic supports. Therefore, the subsequent deposition of an ultra-thin but fully dense palladium film becomes more difficult [14], and many researchers opt to modify these metal supports by incorporating inert inorganic particles that partially cover the biggest original pores and smooth the surface before palladium deposition, forming an intermediate layer between the support and the H_2_-selective film [15]. Moreover, this additional layer also serves to prevent any intermetallic diffusion from steel to palladium during operation at high temperatures [16]. The mechanical integrity of the resulting composite membranes highly depends on the nature of these particles and the thermal compatibility between the materials of each stacked layer of the membrane. In this context, CeO_2_ particles appear to represent an excellent alternative due to their thermal expansion coefficient, very similar to that exhibited by palladium and steel in the range of 12–13 μstrain/°C [12,17,18]. 

The incorporation of a H_2_-selective film onto the external surface of tubular porous supports prevails in the literature, probably intending to facilitate its morphological characterization through non-destructive techniques and avoid the membrane delamination produced in most applications where the permeated pure hydrogen is collected on the lumen side [19,20]. However, these membranes can also present diverse operating problems that limit their performance, especially in the case of working with complex gas mixtures [21,22,23] or fluidization conditions [24,25]. In this context, certain gases, such as carbon monoxide or steam, can inhibit the permeation process through the palladium film, especially above a certain concentration in the feed mixture [23,26], while fluidized catalyst particles could damage the palladium film by collisions, thus facilitating the generation of cracks and defects [24,27]. As a consequence, the total permeate flux and H_2_-selectivity usually decrease under these conditions [28]. These drawbacks can be limited by incorporating an additional protective layer directly onto the palladium surface made of any porous material, thus avoiding the direct impact of fluidized catalyst particles on the metal or reducing the amount of inhibiting compounds in the neighborhood of the H_2_-selective film. This strategy was followed by Arratibel et al. [25,29], incorporating an additional Al_2_O_3_–YSZ porous layer of around 1 μm onto a Pd–Ag film, previously deposited over a Hastelloy X tubular porous support, thus reaching a good maintenance stability of virtual infinite ideal perm-selectivity of the membrane even after long-term tests under fluidization conditions. However, these authors also note some possible problems for these membranes, especially related to the delamination of the above-mentioned protective film during long-term tests. This observation could be produced by its limited adherence and the appearance of certain mass transfer limitations in mixture conditions. A similar strategy was adopted by Guo et al. [30] by incorporating a thin TS-1 zeolite film on a palladium membrane, demonstrating good stability of the new membrane for 10 days of hydrogen permeation at 400 °C and gas exchanging tests, thus preventing any hydrocarbon contamination of the selective film. On the other hand, Zhao et al. [31] prepared a composite membrane in which two different Pd layers were deposited on both sides of a porous ceramic tube, external and internal, to eliminate the mass transfer resistance resulting from sweep gas diffusion into the support of other conventional asymmetric composite membranes. As a result, with an equivalent total palladium thickness in comparison with other traditional supported membranes, the new alternative provided an enhancement of an order of magnitude in the H_2_-selectivity but a similar H_2_-permeance.

Despite all the above-mentioned alternatives, considered to prevent some of the most relevant operating problems of composite membranes, the direct use of the ceramic intermediate layer placed on the surface of porous supports for protection purposes of the Pd film is scarce in the literature. Besides the intrinsic difficulties in characterizing these samples through non-destructive analyses at a lab-scale, the mechanical resistance of the resulting composite material could play a critical role. In this context, the palladium incorporation onto modified PSS supports through the electroless pore-plating (ELP-PP) technique developed by Alique et al. [32] demonstrated an excellent adherence to the porous substrate under diverse operating conditions and long-term permeation analyses, even in the case of generating tensile stresses due to the particular permeation flux direction [14,33,34]. In this manner, the excellent mechanical resistance of composite membranes prepared by ELP-PP was observed independently of collecting the permeate flux from the porous support side or the contrary one, where the Pd-film is incorporated. On this basis, the current study aims to delve deep into the versatility of ELP-PP membranes, fabricating and testing new composite membranes in which both the ceramic intermediate layer and Pd-film are placed on the internal surface of tubular PSS supports instead of the external one, as is usually done. This type of membrane, published here for the first time, could present a new attractive alternative for particular applications in which the Pd-film needs to be protected (i.e., fluidized membrane reactors) while avoiding the incorporation of additional layers that increase the complexity of the membrane and could compromise their lifespan. 

## 2. Materials and Methods

### 2.1. Membrane Preparation

**Figure 1:**
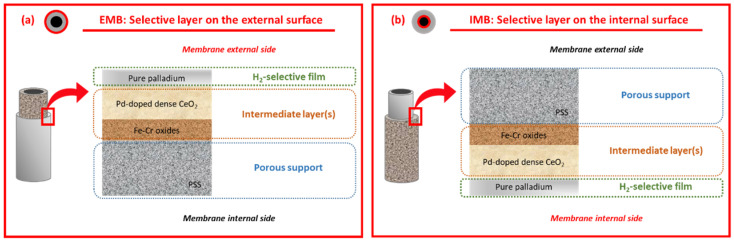
Schematic structure of composite membranes: selective layer on the external (**a**) or the internal (**b**) surface or tubular PSS supports.

For the membrane preparation, symmetric 316L porous stainless steel (PSS) supports from Mott Metallurgical Corp. (Farmington, CT, USA) with cylindrical geometry were used. The original supports have an average porosity of ca. 20% and a media grade of 0.1 μm, which implies that 95% of the solid particles greater than this value can be rejected. These porous supports, with an external diameter of 12.9 mm, have a wall thickness of 1.9 mm. The original tubes were cut into smaller pieces of around 30 mm in length before starting the synthesis procedure. After that, the general preparation procedure includes the next successive steps: initial cleaning, calcination in air, incorporation of a ceramic intermediate layer, and Pd deposition by electroless pore-plating (ELP-PP). Despite maintaining a common fundamental composite-membrane structure based on diverse stacked layers (i.e., Fe–Cr oxides, CeO_2,_ and palladium), its particular conformation varies according to the position of the Pd-film, on the external or internal surface of the tubular PSS support. In this context, Figure 1 schematically illustrates both alternatives, denoting the membranes as EMB or IMB in the case of incorporating the layers on the external or the internal surface of original supports, respectively. 

Focusing on the membrane fabrication process, the first cleaning step involves consecutive washings in sodium hydroxide 0.1 M (5 min), hydrochloric acid 0.1 M (5 min), and ethanol 96% vol. (15 min). In all cases, the cleaning was performed at 60 °C in an ultrasonic bath with intermediate rinsing in deionized water. Then, clean supports were dried overnight at 110 °C, and their weight was taken as reference for the subsequent preparation steps. These supports were then calcined in air at 600 °C for 12 h with a heating rate of 1.0 °C/min to generate a first intermediate barrier between the PSS support and the Pd film made of Fe–Cr oxides coming from the steel [35].

Next, an additional ceramic intermediate layer, formed by doped CeO_2_ particles with Pd nuclei, was incorporated via pressure-assisted dip-coating (PA-DC) to fabricate the composite membrane in which the selective films are placed on the inner side (IMB, Figure 1b). For this purpose, a suspension was prepared to contain commercial CeO_2_ particles with an average size of around 100 nm from Alfa-Aesar (Haverhill, Massachusetts, USA) (20 wt.% load) and polyvinyl alcohol (PVA) from Sigma-Aldrich (San Luis, Missouri, USA) (2 wt.% load) in deionized water, incorporating the ceramic material on the internal surface of the membrane by PA-DC. Particularly, silicon sealing O-rings were used to isolate both the internal and external sides of the membrane during the procedure. The suspension was introduced from the inner side of the membranes by applying pressure (0.50 bar) during the last two minutes of the second immersion to guarantee the homogenous incorporation of the ceramic particles into the largest pores of the support. After that, the modified supports were calcined at 500 °C for 5 h to ensure their stability by removing the PVA from the intermediate layer. For comparison, a similar procedure was considered to generate the CeO_2_ intermediate layer on the external surface of the calcined supports (EMB, Figure 1a) instead of the internal one, as previously reported [36,37]. The main difference, apart from the side on which the suspension was placed, was the generation of a vacuum from the lumen side of the membrane to facilitate the incorporation of the particles inside the pores rather than applying pressure from this side as previously described. In both cases, independently of incorporating the intermediate layer on the inner or the outer surface of the membrane, the commercial CeO_2_ particles were superficially doped with fine palladium nuclei before their incorporation, denoting the new material as Pd–CeO_2_ particles. For that, raw CeO_2_ particles were vigorously stirred at room temperature into a suspension of the particles with a volume ratio of 1:18 related to a solution containing 0.1 g/L PdCl_2_. Then, a certain amount of hydrazine 0.2 M was added into the suspension to provoke the reduction of palladium ions into metal nuclei, which are deposited on the external surface of the CeO_2_ particles. Finally, once the intermediate layers were incorporated on the porous supports, the palladium was deposited by ELP-PP according to the experimental procedure detailed elsewhere [34,36,37]. Briefly, two solutions containing the Pd source and the reducing agent were placed on opposite sides of the porous substrate to force the chemical reaction between them either just in the pores or around their neighborhood. For this last step, it is important to point out that diverse recurrences are necessary to ensure the generation of a fully dense membrane. This was reached when the membrane weight gain after a particular recurrence became negligible, thus suggesting a complete sealing of the pores with palladium.

In this context, Table 1 collects detailed information about the particular composition for the solutions considered in both activation and plating steps, as well as the particular operating conditions for each case.

### 2.2. Membrane Characterization

#### 2.2.1. Morphological Characterization

In all samples, the morphology reached after each previously described experimental step was completely characterized by different techniques. First, the total amount of materials incorporated onto the substrate (Fe–Cr oxides, CeO_2_, and palladium) was determined by the weight gain with the help of an electronic balance Kern & Sohn ABS-4 (Balingen-Frommern, Germany) with a precision of ± 0.0001 g. These values, especially those related to the incorporation of palladium by ELP-PP, were used to estimate the average thickness of the metal film. Then, the surface morphology of the samples was additionally evaluated by using scanning electron microscopy (SEM, Hitachi S-2100N microscope equipped with an energy dispersive analytical system for microprobe analysis -Tokyo, Japan-). Digital Micrograph^®^ software (Pleasanton, CA, USA) was used for image segmentation and the quantification of average pore size distribution on the surface of PSS supports before and after the incorporation of intermediate layers. Besides the SEM analyses on the membrane surface, the real thickness for the reached palladium film was also measured through cross-sectional views after cutting and polishing the membranes in annular sections after being completely tested in permeation.

#### 2.2.2. Permeation Measurements

**Figure 2:**
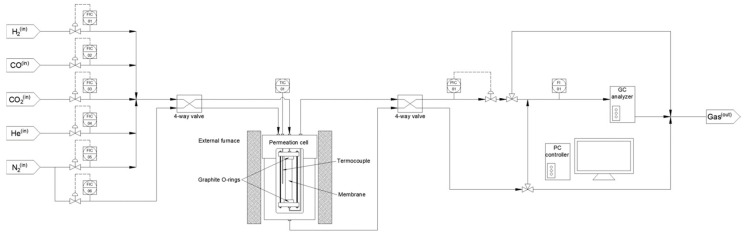
Schematic representation of the equipment used for permeation experiments at high temperatures.

First, some preliminary leak tests in pure ethanol at room temperature were performed to ensure the absence of defects in the palladium film. The test consists of feeding helium at pressures up to 3 bar, observing the potential generation of bubbles on the membrane surface caused by the permeation of the gas through eventual defects in the palladium films. In this context, the absence of bubbles under these conditions suggests a good continuity and density of the metal film for further experiments. Hence, the detailed permeation behavior of the membranes exhibiting a complete helium tightness was determined for higher temperatures and diverse gas compositions in an experimental setup described in detail in previous works [38,39]. Figure 2 collects a schematic representation of the permeation system used for these tests. The permeator is based on a stainless steel cell in which the Pd-membrane is placed between two graphite O-rings to ensure a good sealing between the retentate and permeate sides. The assembly is heated by an external electrical furnace to achieve the desired temperature for each experiment (350–450 °C). Pure gases (nitrogen, helium, carbon dioxide, carbon monoxide, and hydrogen) can be fed individually or together to prepare concrete mixtures thanks to various Bronkhorst Hi-Tech mass-flow controllers (Ruurlo, The Netherlands) with a maximum capacity of 400 NmL min^−1^ mounted in line. The pressure difference between the retentate and permeate sides was varied from 0.5 to 4.0 bar to analyze the influence of diverse permeation driving forces by maintaining atmospheric pressure on the permeate side and controlling the retentate pressure with a Bronkhorst High-Tech EL-PRESS back-pressure regulator (Ruurlo, The Netherlands). In all cases, the permeation behavior was evaluated from the permeation fluxes (measured with a Horiba precision soap film flow-meter -Kyoto, Japan- with minimum and maximum capacities of 0.2 and 10^4^ mL min^−1^, respectively), resulting permeability, and hydrogen selectivity. No sweep gas was used on the permeate side for the entire set of experiments. Moreover, a gas chromatograph (GC), Varian CP-4900 (Santa Clara, California, USA), with a thermal conductivity detector (TCD) and two analytical columns (Molsieve 5A and PoraPLOT-Q) was mounted in the outlet line to analyze the particular composition of the permeate or retentate streams. Finally, it should be noted that feed streams throughout the entire study, independently of considering pure gases or mixtures, were always introduced from the inner side of the tubular membrane, thus concentrating the permeate stream on the shell side.

## 3. Results and Discussion

This section is divided into several sub-sections, each of which focused on a particular aspect related to the characterization of the prepared membranes. First, the evolution of the membrane after each successive preparation step is discussed, comparing the resulting morphology with the analogous one reached in previous studies in which the selective layer was placed onto the external side of tubular supports. Then, a series of permeation tests performed at different operating conditions are included to analyze the permeation behavior of the membranes. In this context, it should be noted that preliminary leakage tests in ethanol with helium were made to ensure the absence of other gases different from hydrogen in the permeate, thus ensuring a high perm-selectivity of the membranes.

Once this aspect was confirmed, experiments with pure gases, nitrogen, and hydrogen, were carried out to characterize the intrinsic membrane performance, understanding the term “intrinsic” as the potential interactions of the membrane with the key-species scope of the investigation, i.e., hydrogen.

These results were also completed with further permeation experiments in which diverse binary gas mixtures were fed to the membrane in analogous operating conditions of temperature and pressure.

### 3.1. Membrane Morphology

**Figure 3:**
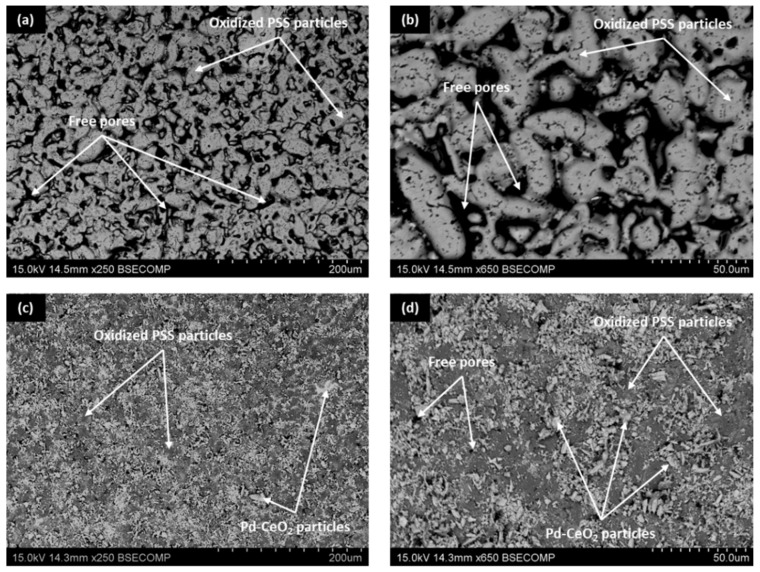
SEM images of PSS tubes after the incorporation of internal intermediate layers at diverse magnification: Fe–Cr oxides by calcination in air (**a**,**b**) and Pd–CeO_2_ particles by PA-DC (**c**,**d**).

First, the morphological evolution of the PSS internal surface to modify both the original roughness and pore sizes by the incorporation of diverse intermediate layers was characterized by SEM analyses, producing some of the most representative images in Figure 3.

As can be seen, a large number of irregular pores typical in PSS remain after the initial calcination step in air at 600 °C (Figure 3a,b). A detailed analysis by image segmentation of both the porosity and average pore size after the support calcination revealed values of around 10% and 2.2 μm, respectively. However, it was still possible to find some pore mouths larger than 15 μm that could compromise the formation of a fully dense Pd-film of a few micrometers. These values are quite similar to those obtained on the external surface of analogous PSS supports after their calcination in air, and only represent a slight reduction compared with uncalcined ones [35]. Therefore, it can be stated that the generated oxide layer around each stainless steel particle was thin enough to avoid a drastic modification of the original surface morphology on the internal and external sides and further modifications are required to fabricate composite membranes with limited palladium thickness. In this context, the subsequent incorporation of Pd–CeO_2_ particles by PA-DC partially blocked most of the largest pores present in calcined PSS supports (Figure 3c,d), generating a new porous structure in which a relatively narrow distribution of pore-mouth sizes was reached. In fact, the largest pore mouths of the new surface remained below 3.5 μm with an average pore size of around 0.8 μm. In this manner, it was expected to obtain a thinner and fully dense Pd-film with a good continuity onto the new morphology, as suggested in previous works by Shi et al. [40] and Yepes et al. [41], among others.

**Figure 4:**
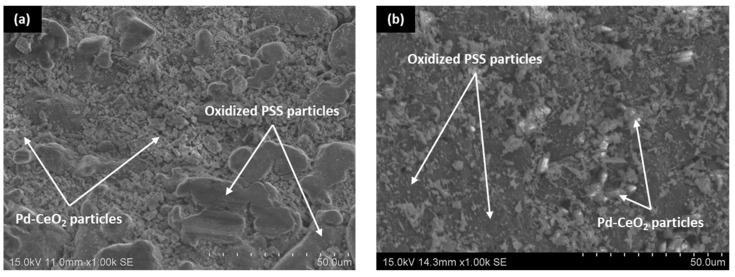
SEM micrographs of Pd–CeO_2_ intermediate layer reached on contrary sides of calcined PSS supports: (**a**) external and (**b**) internal.

In this context, for comparison purposes, Figure 4 collects the surface reached just after the incorporation of the ceramic particles by dip-coating onto both internal and external surfaces of calcined PSS supports. It should be highlighted that the intermediate layer generated on the internal side of the porous supports is presented in the current study for the first time, while its incorporation onto the external surface has been typically considered in other previous works [36,37]. Regarding the experimental incorporation procedure, the main differences are based on how the ceramic particles are forced into the pores of the substrate. Thus, a vacuum from the inner side of the supports is used to facilitate this task in the case of incorporating the ceramic intermediate layer onto the external surface of porous supports, in contact with the dip-coating suspension, while pressure from the lumen side, previously filled with the above-mentioned suspension, is considered to incorporate the intermediate layer onto the internal surface of PSS supports. In both cases, after completing two consecutive recurrences of dip-coating for better homogeneity, the ceramic material in excess is carefully removed in wet conditions as detailed in previous work [36]. In general, the ceramic particles incorporated in both cases mainly take up all deeper areas related to the largest pore-mouths of porous substrates, although certain differences can be appreciated in terms of the total amount of ceramic particles incorporated as intermediate layer per membrane area. Thus, around 1.65 × 10^−3^ g cm^−2^ are deposited onto the external surface, while this amount was slightly increased up to 1.70 × 10^−3^ g cm^−2^ when the modification was carried out on the internal surface of the support. In general, good reproducibility can be assumed, attributing the small differences to the particular procedure followed during the ceramic excess removal after the dip-coating. Most likely, the procedure is more difficult to perform with precision from the inner side of the tubular support. Despite the slight deviations in the total amount of incorporated material for each alternative, the image segmentation suggests similar modifications with deviations below 4% in both average porosity and mean pore size.

**Figure 5:**
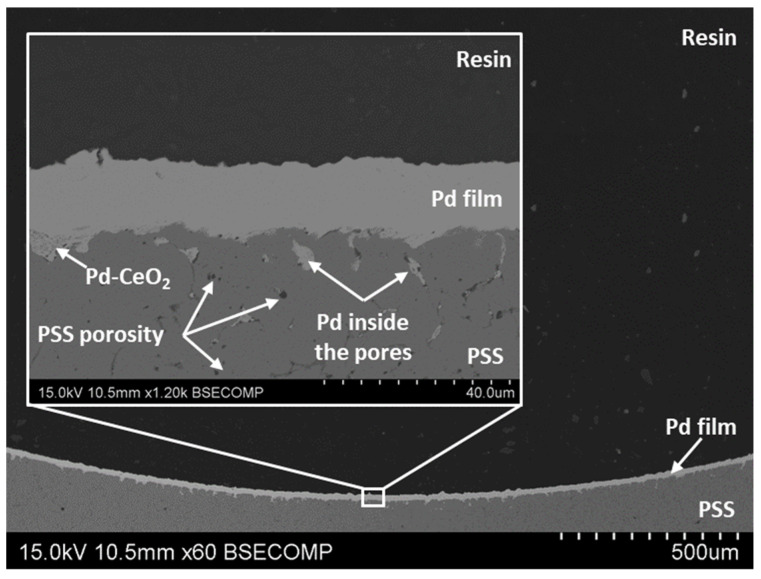
SEM cross-sectional view of ELP-PP membrane prepared onto modified supports with doped CeO_2_ intermediate layer.

After analyzing the morphology of modified PSS supports with both Fe–Cr oxides and Pd–CeO_2_ intermediate layers, the most relevant characteristics of the Pd-film incorporated by ELP-PP onto the internal surface have also been addressed. In this context, Figure 5 illustrates the cross-sectional view of the reached composite membrane with a traditional sandwich-type structure. As previously discussed, most of the largest pores near the top surface are filled with Pd–CeO_2_ particles and, similar to other ELP-PP membranes, the palladium is distributed in two well-differentiated areas: (i) a top film onto the internal surface of modified PSS tube and (ii) just inside the remaining free-pores nearest to this surface [33]. This fact can be explained by considering the nature of the ELP-PP process, in which both solutions, metal source and reducing agent, are fed from opposite sides of the porous support, initiating the chemical reaction between them just inside the pores or around their neighborhoods. However, the reducing agent (hydrazine solution) can easily pass through the largest pores towards the other solution containing the palladium, thus provoking the generation of an almost continuous top film on this side. Keeping the same supports, ceramic particles for the generation of the intermediate layer and solutions for the palladium incorporation by ELP-PP, certain differences can be appreciated when comparing composites membranes obtained when the H_2_-selective film is placed on the external or on the internal surface, respectively. In general, an average thickness of around 10–12 μm, estimated from gravimetric analyses, was reached in the case of incorporating both intermediate layers and Pd film onto the external surface of tubular PSS supports, as reported in previous studies [36,37].

Typically, the real thickness of the top Pd film was even lower than these values due to a certain amount of palladium placed just inside the pores, not in the top coating as considered in the preliminary estimation. Nevertheless, as can be appreciated in Figure 5, in the case of generating these layers on the internal side of the tubular substrates, a real Pd-thickness slightly higher, with values in the range of 12–15 μm, was reached. Moreover, it should be noted that the new palladium film does not replicate the original PSS surface morphology, as typically occurs when analogous films are deposited on the external side [37]. This can be explained by differences in the final Pd-thickness, probably due to the variation in the volume ratio between ELP-PP solutions containing both a metal source and reducing agent. Particularly, this ratio was maintained around 16:1 during the generation of the Pd film on the external surface but changed to 14:1 in the contrary case when incorporating the Pd film on the internal PSS surface, mainly due to restrictions of the static experimental setup (available volume into the membrane lumen and minimum liquid level for ensuring a complete immersion of the membrane). These results suggest that the further optimization of these parameters could be considered for reducing the thickness of these internal films.

### 3.2. Permeation Behavior in Pure-Gas Conditions

The permeation behavior of this kind of membrane, in which a Pd-film is incorporated on the internal surface of tubular PSS supports, was first discussed in the case of feeding pure gases. Initially, the gas tightness of the composite membrane was evaluated with helium at room temperature, thus ensuring the good continuity of the Pd film and complete blockage of the original support porosity. These preliminary results were later corroborated through a set of experiments carried out by feeding pure nitrogen at higher temperatures within the range of 350–450 °C. As a result, no nitrogen in the permeate side was observed at pressures in the range of 0.25–4 bar (according to the detection limit of the flowmeter, 1.67 × 10^−2^ mL min^−1^), thus suggesting an eventually complete ideal separation factor. At this point, the particular permeation behavior was studied after switching the feed stream to pure hydrogen and maintaining analogous operating conditions. In all cases, the permeate pressure was maintained at the atmospheric one without applying any sweep gas.

**Figure 6:**
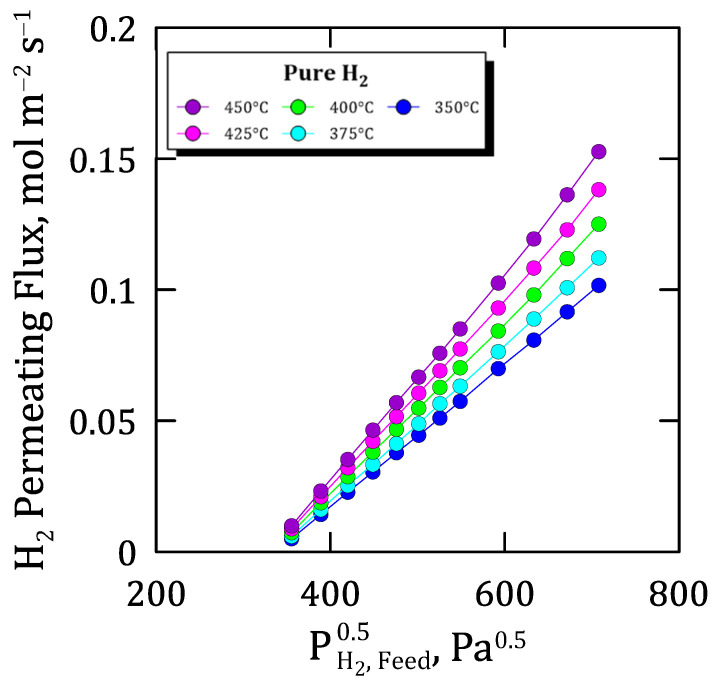
General permeation behavior of the membrane at diverse temperatures and pressures while feeding pure hydrogen.

The obtained results under these conditions are summarized in Figure 6, where the permeating flux is plotted against the square root of the hydrogen partial pressure on the feed side. First, it should be noticed that, at higher pressure and temperature values, the flux profiles reveal a tendency to be more than linear, probably due to a non-ideal hydrogen permeation caused by the relatively high concentration in the metal lattice. However, this deviation is so slight within the selected range of pressures that the ideal permeation of hydrogen through the membrane can be assumed with a minimum error, as suggested by Caravella et al. [42].

**Figure 7:**
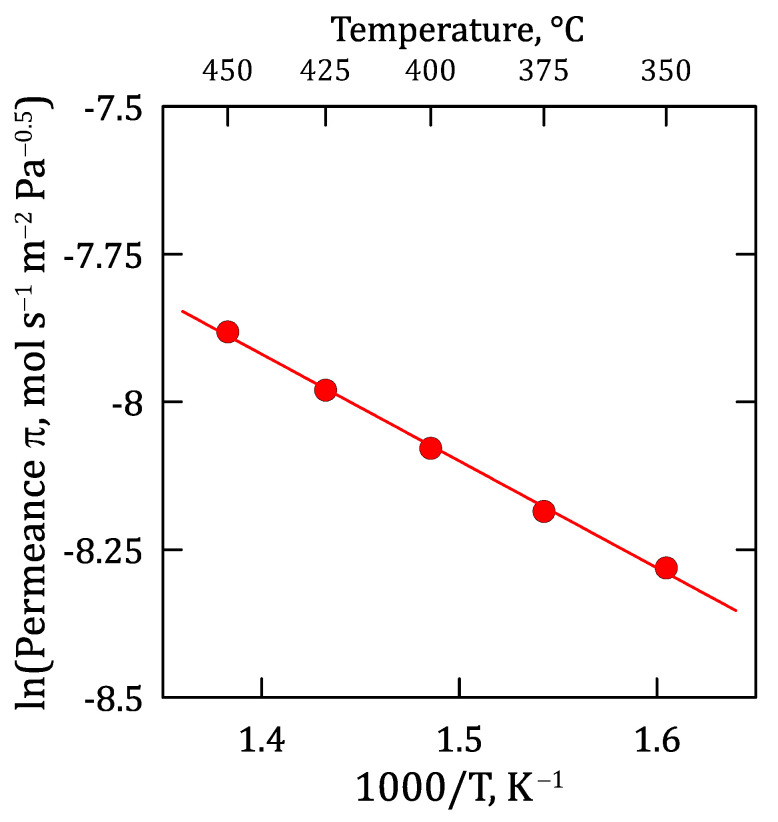
Arrhenius-type plot for evaluating the temperature influence on H_2_ permeation.

The detailed influence of operating temperatures during permeation experiments is shown in Figure 7 through a traditional Arrhenius-type plot, in which the H_2_-permeance is represented against the inverse of temperature. As usual, higher temperatures increase the reached H_2_-permeance through the composite Pd-membrane with a good linearity between both parameters, as evidenced by the satisfactory R-squared coefficient (R^2^ = 0.998). The goodness of fit measure for the linear regression suggests that the approximation made while considering Sieverts’ law within the experimental temperature range is reasonably acceptable.

Table 2 reports the most representative data about the permeation behavior in pure-gas conditions of the membrane presented in this work in comparison with other relevant ones from the literature. The wide variety of structural designs, morphological properties, and operating conditions in permeation tests, among other factors, certainly make rigorous comparison difficult. In this context, for a more specific and useful comparison, only composite membranes prepared by electroless plating or related techniques onto tubular PSS supports were selected. For these cases, details about the intermediate layer composition, the most relevant characteristics of the H_2_-selective film, and the membrane performance are included in the table.

First, it should be emphasized that most of Pd-films are incorporated onto the external surface of the porous supports, just on the contrary side to those considered in the present study. This could be explained by a greater facility to characterize the composite membrane by non-destructive techniques but also by the configuration of permeation setups, in which it is usual to permeate from the outer to the lumen of the membrane, thus avoiding possible delamination of the palladium film if it is placed solely on the external surface. In general, the thinnest selective films allow the highest hydrogen permeation. At this point, it is necessary to remark that a very high permeance is usually related to a limited H_2_ selectivity of the membrane, thus permeating not only through the metal lattice by solution-diffusion mechanisms but also through membrane defects by Knudsen diffusion. Higher permeation is also typically reached when the H_2_-selective film is made of alloys containing silver, copper, or gold instead of pure palladium. A wide variety of values for activation energy are observed for each particular membrane, although most of them are maintained in the range of 8–16 kJ mol^−1^. This range is within the typical one reported for dense palladium membranes in the literature, independent of the fabrication method. More deviations can be found when analyzing the ideal H_2_/N_2_ separation factor, with values from around one hundred to infinite. The membranes fabricated for this work exhibited values higher than 10,000 for the entire set of experiments, thus being placed at the top of the list. This fact belies the excellent mechanical resistance of the ELP-PP membrane fabricated on the internal surface of the tubular porous support, it being difficult to find a similar arrangement in the literature where the Pd-film is deposited onto the internal surface of tubular supports. A comparison of the particular permeances and activation energies reached for the membranes fabricated for the current study with other membranes in which the Pd film is incorporated on the external surface of the tubular supports revealed no significant differences. This fact demonstrates the ability of ELP-PP technology to reach composite membranes with a highly reproducible performance, independently of incorporating selective layers on the external or internal surface of tubular substrates. 

### 3.3. Permeation Behavior in Binary Mixtures

In addition to permeation tests with pure gases, the membrane performance was also analyzed for permeation with binary mixtures in which hydrogen was fed together with different non-permeating gases (N_2_, He, CO_2_ and CO) at diverse concentration values (0, 10, 20, 30, 40, and 50% vol.) and temperatures (350–450 °C) to investigate the influence of possible mass transfer resistances generated by the porous support itself, the concentration polarization effect, or potential inhibition effects of certain non-permeating compounds.

#### 3.3.1. H_2_–N_2_ Mixtures

**Figure 8:**
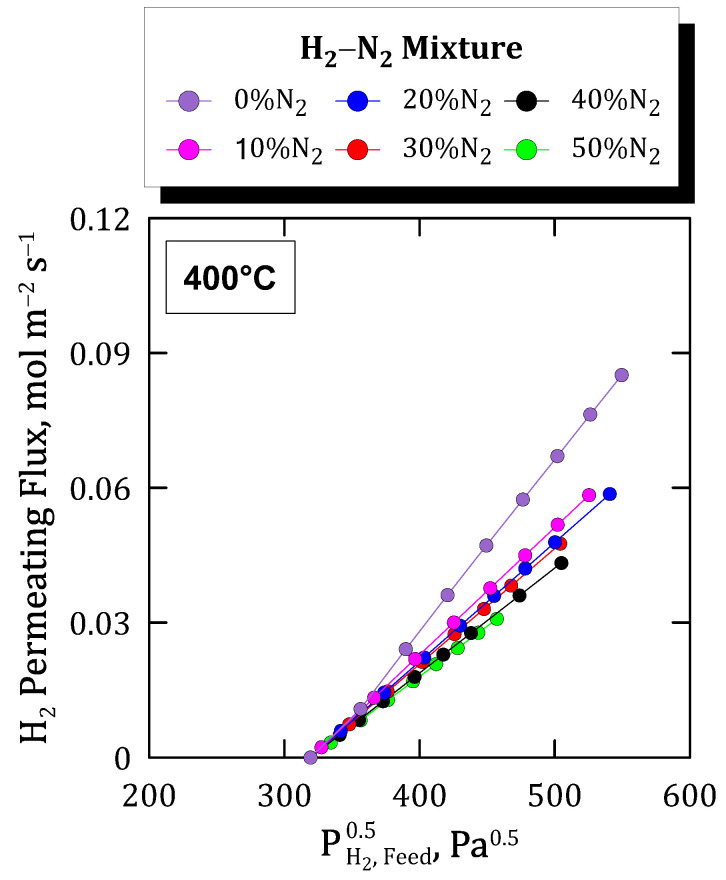
Hydrogen permeating fluxes for H_2_–N_2_ binary mixtures at different feed compositions.

First, the analysis of permeation behavior when feeding binary H_2_–N_2_ mixtures, evaluated as individual gases in the previous section, is addressed. In this context, Figure 8 collects the permeating fluxes reached at 400 °C as a function of the square root of the hydrogen partial pressure in the feed. Each data series collects diverse nitrogen compositions in the feed while maintaining analogous pressure driving forces for each particular point.

In this context, the permeate flux decreases with increasing nitrogen composition, which could be expected due to progressively higher mass transfer resistances. However, this difference was found to be lower with diluting the feed stream and, hence, increasing the N_2_ composition. This suggests that the increase in the external mass transfer resistance was not linear with the composition of the non-permeating species, particularly with the non-inhibiting ones, such as nitrogen. It is also important to notice that no nitrogen was found through gas chromatography analyses on the permeate side.

**Figure 9:**
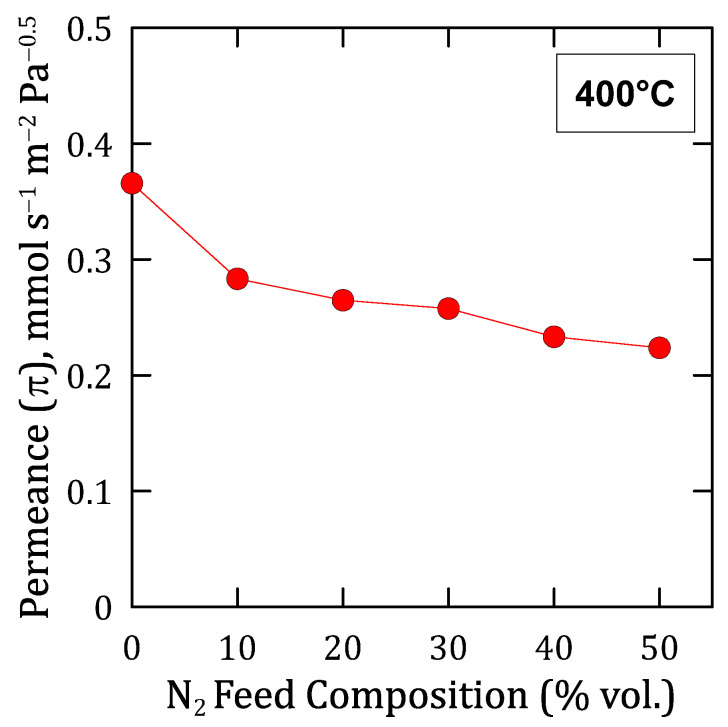
Hydrogen permeance at 400 °C reached for different compositions of H_2_–N_2_ mixtures.

On the other hand, it should be noticed that all the flux trends were approximately linear, which allows us to evaluate precisely a Sieverts-type permeance for each composition. The evolution of these permeance values given under each particular condition is shown in Figure 9. As it can be seen, a continuous decreasing trend was produced for increasing nitrogen concentrations in the feed, being especially marked when comparing the permeation reached by feeding pure hydrogen with a binary N_2_–H_2_ mixture, even in the case of a relatively low nitrogen concentration. This behavior is typical in other similar Pd-based membranes and can be explained by the concentration-polarization effect described in multiple studies [28,51,52,53]. This effect, which negatively affects the overall permeation through the membrane, is a natural consequence of the high selectivity of the composite membrane. In this manner, the non-permeating species, nitrogen in this particular case, is accumulated onto the feed side of the Pd film, thus increasing its concentration at the boundary layer close to the palladium surface due to the selective transport of hydrogen through the precious metal [54]. This phenomenon, here evidenced for H_2_–N_2_ binary mixtures, will always occur for any other gas mixture, although its relevance could vary as a function of the particular properties of compounds present in the mixture, mainly their kinetic diameters. Moreover, the presence of a porous structure close to the surface, i.e., considering the use of any porous support, intermediate layer, or porous protective film for the palladium film, surely increases this phenomenon. 

#### 3.3.2. H_2_–He and H_2_–CO_2_ Mixtures

**Figure 10:**
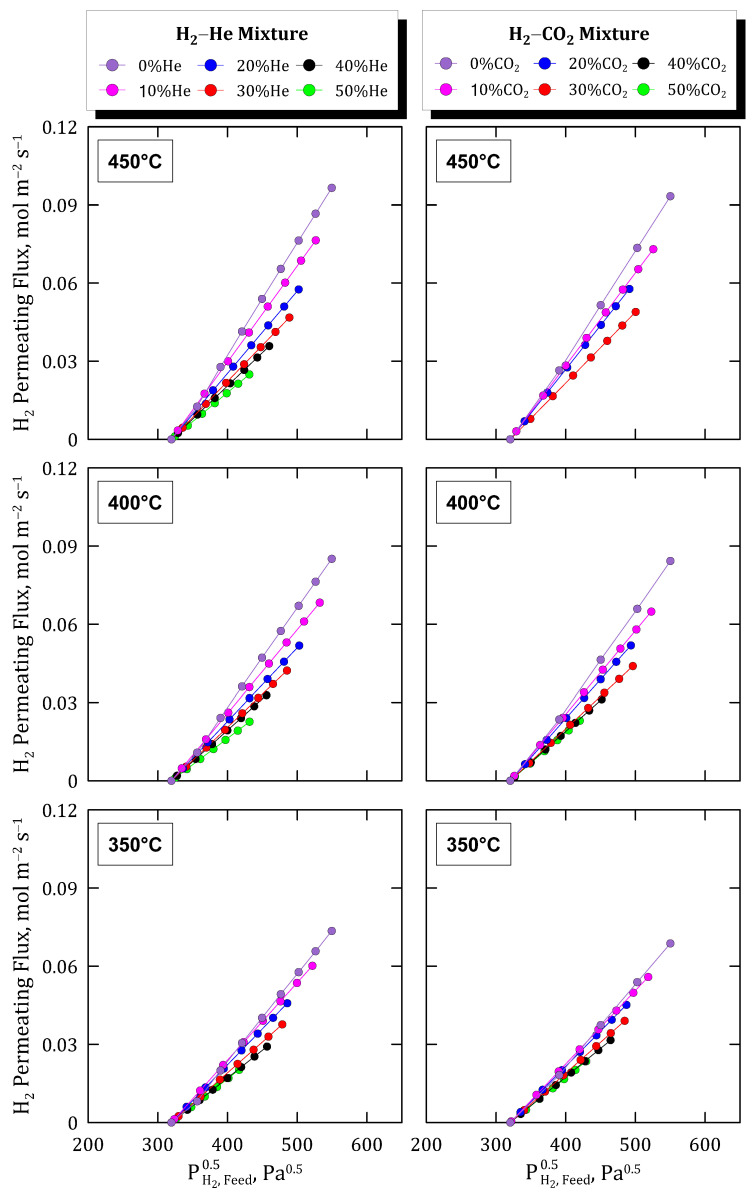
Hydrogen permeating fluxes reached at different temperatures and feed concentrations in H_2_–He and H_2_–CO_2_ binary mixtures.

Considering the interest in elucidating possible differences while testing binary mixtures of hydrogen with other inert molecules of diverse kinetic diameters, as suggested in the previous section, new experimental campaigns were performed. These tests were similar to those previously addressed, although the amount of nitrogen (d = 3.64 × 10^−14^ m) in the mixture was replaced by helium (d = 2.60 × 10^−14^ m) or carbon dioxide (d = 3.30 × 10^−14^ m), both molecules with lower kinetic diameters. In addition, the experiments were performed at three different temperatures in the range of 350–450 °C to better analyze the membrane performance in different changing conditions. All the results obtained from these experimental campaigns are collected in Figure 10, where the permeation fluxes reached for a particular binary mixture are organized in columns while rows are dedicated to each particular temperature considered during the tests.

First, it should be noted that, according to the gas chromatography measurements, only hydrogen can be detected in the permeate stream, thus maintaining a membrane of excellent selectivity. Moreover, analyzing the trend given by the experimental data, all the experiments provide, in general, a reasonably good linear fit between hydrogen flux in the permeate and the square root of hydrogen partial pressure. This suggests, once again, the validity of Sieverts’ law for predicting the permeation fluxes through the membrane in the considered ranges of operating conditions.

Focusing on the permeation data obtained at 400 °C and considering the above-mentioned results when feeding H_2_–N_2_ mixtures as a reference, an increase in the permeation fluxes for both H_2_–He and H_2_–CO_2_ mixtures can be observed. In the first case, this effect can be justified by the higher diffusivity of helium over nitrogen due to its lower molecular kinetic diameter, thus partially reducing the concentration-polarization effect on the membrane. A similar effect was appreciated in the case of testing H_2_–CO_2_ mixtures, reaching slightly higher permeate fluxes in comparison to the experimental campaign carried out with H_2_–N_2_ mixtures. In the last case, the differences were lower due to the similarity of kinetic diameters between nitrogen and carbon dioxide. The similarity between all the measurements suggests that the mass transport through the membrane is driven by the transport in the metal layer and, hence, the external resistance to permeation is just slightly appreciable and provides a relatively small contribution to the overall process.

Attending to the temperature effect, similar trends can also be appreciated for both cases. The concentration-polarization effect given by the presence of non-permeating species in the feed mixture appears in all cases, independently of the particular compound, helium or carbon dioxide, and temperature value. However, the relevance of this negative effect on the permeation capacity of the membrane seems to be promoted by increasing temperatures. In fact, as previously addressed for pure gases, higher temperatures increase the permeation rate as suggested by the typical Arrhenius-type dependence expression. As a consequence, the concentration of non-permeating species close to the side of the palladium film increases, thus making more evident the negative contribution of the concentration-polarization effect in the overall permeation behavior.

**Figure 11:**
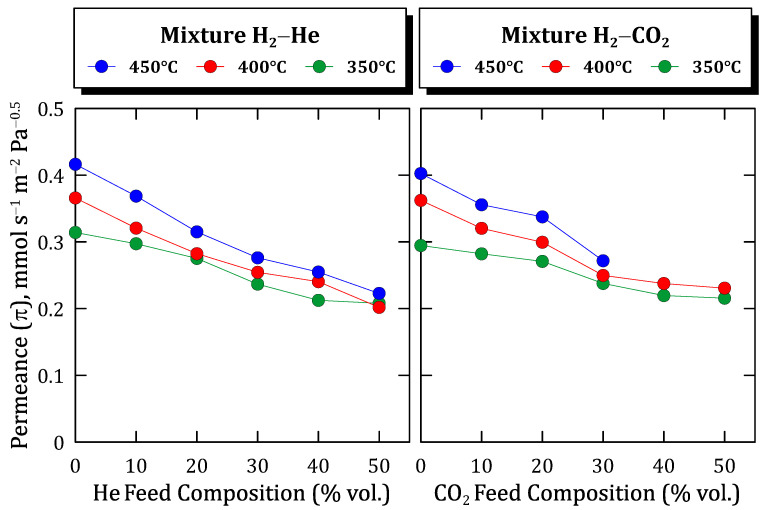
Hydrogen permeance against feed composition at different temperatures.

To analyze these preliminary conclusions more clearly, Figure 11 collects all the experimental data for both the H_2_–He and H_2_–CO_2_ binary feed mixtures. In the figure, the evolution of the calculated permeance values at each particular experimental condition, assuming the validity of Sieverts’ law, is depicted. 

As can be seen, in all cases a very similar descending tendency was obtained, except for the points calculated for a concentration around 20% vol. of the non-permeating species, in which a certain deviation can be appreciated probably due to the typical experimental uncertainty. The above-mentioned effects related to feed composition and temperature are clear. The permeance decreases for increasing dilution grades in the feed stream while increasing for higher temperatures. In this context, the concentration-polarization effects become more relevant in the case of higher permeation fluxes. Finally, it should be noted that a great similarity between values was reached by considering mixtures containing different non-permeable and inert species.

#### 3.3.3. H_2_–CO Mixtures

**Figure 12:**
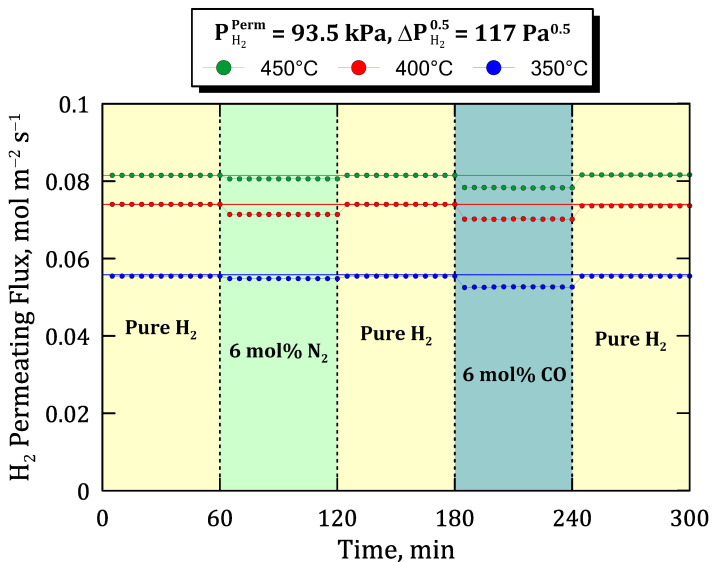
Time profile of hydrogen permeating flux at different temperatures with feed mixtures containing 6 mol% of N_2_ or CO ($P_{H_{2}}^{Perm}$ = 93.5 kPa, $\Delta P_{H_{2}}^{0.5}$ = 117 Pa^0.5^).

Previous experiments, in which binary mixtures containing relatively inert species were fed to the system, can be completed with other ones that could generate some inhibition, as occurs with carbon monoxide as widely reported in the literature [23,26,28]. To assess the potential inhibition effect produced by CO, the time profiles of permeate hydrogen flux are collected in Figure 12 for a specific hydrogen partial pressure on the permeate side ($P_{H_{2}}^{Perm}$ = 93.5 kPa) and a certain value of Sieverts’ driving force ($\Delta P_{H_{2}}^{0.5}$ = 117 Pa^0.5^) but different operating conditions in terms of temperature and feed composition. In this context, three different sets of experiments were carried out at temperatures ranging from 350 to 450 °C. In all of them, first, the system is fed with pure hydrogen, changing the stream each 60 min by adding alternatively 6 mol% of inert (N_2_) and an inhibiting species (in this case, CO). Between each mixture condition, pure hydrogen is tested again to verify the stability of the membrane.

At this point, it should be emphasized that only hydrogen was detected in the permeate side during the entire set of experiments, thus maintaining an almost complete selectivity of the membrane in all the operating conditions. As expected, the drop in hydrogen permeation fluxes owing to concentration-polarization was lower than that caused by the combination of concentration-polarization and inhibition given by the presence of carbon monoxide in the feed mixture. It should be remembered that any non-permeating species also provide a certain concentration-polarization due to the nature of the permeation process, independently of adding any inhibition effect. Under these conditions, with a relatively H_2_-rich mixture in the feed stream, the original hydrogen flux was almost completely recovered by feeding pure hydrogen at all temperatures, even after being exposed to 6 mol% of CO. This demonstrates a certain ability of the membrane to recover its original performance after several cycles even containing a certain amount of inhibition species in the feed stream.

**Figure 13:**
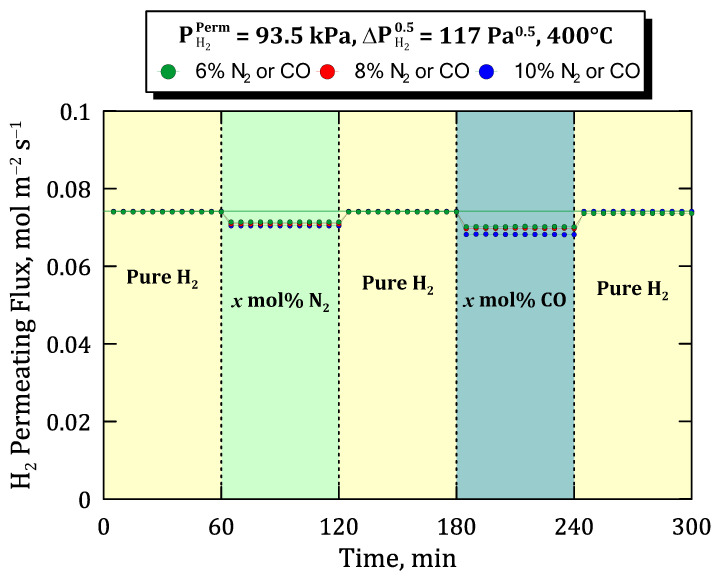
Time profile of hydrogen permeating flux at different concentrations in the feed stream, represented by the symbol “*x*” for each case and maintaining constant T = 400 °C, $P_{H_{2}}^{Perm}$ = 93.5 kPa and $\Delta P_{H_{2}}^{0.5}$ = 117 Pa^0.5^.

To extend the previous study towards more diluted conditions of the feed, similar permeation tests were carried out for different concentrations in the binary mixtures but maintaining constant the temperature at 400 °C, the hydrogen partial pressure on the permeate side in $P_{H_{2}}^{Perm}$ = 93.5 kPa, and Sieverts’ driving force as $\Delta P_{H_{2}}^{0.5}$ = 117 Pa^0.5^ (Figure 13).

Alternative steps, in which pure hydrogen in the feed was replaced by binary mixtures containing inert and inhibition species, were performed, increasing their concentrations progressively from 6 to 10 mol%. As a result, as previously observed, the hydrogen flux was found to be completely recovered by re-feeding pure hydrogen after feeding CO at all the considered composition values and for all the non-inhibiting species used within 60 and 120 min after the start. In this manner, the ability of the membrane to recover its original performance after several cycles containing inhibition species such as carbon monoxide in the feed stream was demonstrated. Moreover, it should be highlighted that all these experiments were carried out for the same membrane without detecting any appreciable decrease in the hydrogen selectivity despite suffering important changes in temperature, pressure, and feed composition operating conditions. This fact guarantees the quality of the membrane and a stable performance for a significant operation time greater than 1000 h.

## 4. Conclusions

New composite Pd-membranes prepared by electroless pore-plating, in which both CeO_2_ intermediate layer and H_2_-selective Pd-film are incorporated onto the internal surface of tubular PSS supports, were satisfactorily fabricated for the first time. No significant differences were found in the morphology reached just after the incorporation of the ceramic particles onto the porous support by dip-coating when compared with the resulting surface on the external side, covering the original largest pore-mouths of substrates. Similar surface morphology was also achieved in the Pd-film, independently of being plated on the external or the internal modified surfaces of PSS tubes, although the average metal thickness required for complete retention of helium and nitrogen was slightly higher, increasing from 10–12 μm to 12–15 μm.

This can be justified by the variation in the volume ratio between ELP-PP solutions containing the metal source and the reducing agent changing from 16:1 to 14:1, and it is foreseeable to prepare more similar membranes after further optimization of the synthesis procedure.

Focusing on the membrane performance, an almost complete ideal separation factor was reached while testing pure gases, also maintaining an excellent H_2_-separation in mixture conditions (π_0_ = 4.571 × 10^−3^ mol m^−2^ s^−1^ Pa^−0.5^ at 400 °C). In general, higher pressure and temperature values increase the permeate fluxes according to Sieverts’ law and Arrhenius-type dependence (E_a_ = 15.031 kJ mol^−1^) with reasonable accuracy. In general, this trend is also maintained for mixture conditions, although the total permeate flux decreases with hydrogen dilution with a non-linear variation in external mass transfer resistance caused by non-permeating species. In this context, the different kinetic diameters of non-permeating species present in the gas mixture also affect the overall permeation fluxes collected during permeation tests. Thus, lower molecular kinetic diameters increase the gas diffusivity and partially reduce the concentration-polarization effect on the membrane. This effect is also dependent on the operating temperature due to higher values which increase the permeation rate and, as a direct consequence, the concentration of non-permeating species close to the palladium film and the concentration-polarization effect. Finally, the potential inhibition effect produced by CO was also evaluated for experiments carried out at diverse temperatures and concentration levels. As expected, the decrease in hydrogen permeation was higher in the presence of CO due to the combination of concentration-polarization and inhibition. However, the original hydrogen flux was almost completely recovered after feeding pure hydrogen for a few minutes, even after being exposed to 10 mol% of CO. This fact ensures the ability of the membrane to recover its original performance after several cycles even containing inhibition species in the feed stream, thus ensuring a stable permeation flux under operation for at least 1000 h.

## Figures and Tables

**Table 1:** Operating conditions and composition in solutions used for both Pd-doping of CeO_2_ particles and Pd-deposition by ELP-PP.

Compounds	Pd-Doping (Activation Stage)	Pd Deposition (ELP-PP)	
		Metal Source	Reducing
PdCl_2_ (g/L)	0.1	5.4	-
NH_4_OH 32% vol. (mL/L)	3.9	390.0	-
EDTA × 2Na (g/L)	-	70.0	-
N_2_H_4_ (mL/L)	0.3	-	10.0
HCl 35% vol. (mL/L)	1.0	-	-
**Operating conditions**			
Temperature (°C)	25	60	
Pressure (bar)	1.0	1.0	
Time (min)	120	1620 ^1^	

^1^ Cumulative time in successive recurrences with cycles of 120 at initial stages and 420 min up to the membrane becomes fully dense.

**Table 2:** Comparison of main characteristics and performance for relevant membranes in literature.

Intermediate Layer	H_2_-Selective Film			Membrane Performance			Ref.
	Incorporation Technique	Composition	*t_Pd_* (μm)	π_0_ (mol m^−2^ s^−1^ Pa^−0.5^)	Ea (kJ mol^−1^)	α_H2/N2_	
YSZ–Al_2_O_3_^(1)^	ELP	PdAg ^(1)^	4–5	2.020 × 10^−3^	5.800	>200,000	[43]
-	ELP	PdAgCu ^(1)^	27	1.667 × 10^−4^	24.500	≈300	[44]
YSZ ^(1)^	ELP	PdAu ^(1)^	4.8	1.179 × 10^−4^	8.200	1000	[45]
YSZ ^(1)^	ELP	PdAuAg ^(1)^	9.3	7.521 × 10^−3^	9.400	500	[45]
Ag ^(1)^	ELP	Pure Pd ^(1)^	6.0	1.489 × 10^−3^	11.900	600–1800	[46]
Al_2_O_3_^(1)^	ELP	Pure Pd ^(1)^	7.0	1.023 × 10^−5^	9.560	92	[47]
Pencil graphite ^(1)^	ELP	Pure Pd ^(1)^	7.0	1.726 × 10^−5^	13.800	≈120	[48]
YSZ ^(1)^	ELP	Pure Pd ^(1)^	25.3	5.700 × 10^−4^	29.200	160–340	[49]
TiO_2_^(1)^	ELP	Pure Pd ^(1)^	14.4	2.600 × 10^−3^	15.370	150–400	[49]
Fe_2_O_3_–Cr_2_O_3_/Pd–SBA-15 ^(1)^	ELP-PP	Pure Pd ^(1)^	7.1	1.847 × 10^−5^	34.600	2550	[38]
Fe_2_O_3_–Cr_2_O_3_/Pd–TiO_2_^(1)^	ELP-PP	Pure Pd ^(1)^	9.7	5.090 × 10^−3^	14.900	∞	[50]
Fe_2_O_3_–Cr_2_O_3_/CeO_2_^(1)^	ELP-PP	Pure Pd ^(1)^	15.4	2.635 × 10^−3^	8.900	≥10,000	[36]
Fe_2_O_3_–Cr_2_O_3_/Pd–CeO_2_^(1)^	ELP-PP	Pure Pd ^(1)^	9.1	6.507 × 10^−3^	13.100	≥10,000	[37]
Fe_2_O_3_–Cr_2_O_3_/Pd–CeO_2_^(2)^	ELP-PP	Pure Pd ^(2)^	13.5	4.571 × 10^−3^	15.031	≥10,000	This work

Location of the intermediate layer and H_2_-selective films: (1) external or (2) internal side of tubular PSS supports.

## Data Availability

Not applicable.
